# Neutrophil gelatinase-associated lipocalin has a stronger association with serum creatinine than C-reactive protein in patients without sepsis; this relationship is lost in septic patients

**DOI:** 10.1186/cc10378

**Published:** 2011-10-27

**Authors:** NJ Glassford, AG Schneider, G Eastwood, L Peck, H Young, R Bellomo

**Affiliations:** 1 Department of Intensive Care, Austin Hospital, Heidelberg, VIC, Australia

## Introduction

Neutrophil gelatinase-associated lipocalin (NGAL) predicts the development of acute kidney injury (AKI) amongst critically ill patients [1]. Serum and urinary NGAL have been shown to be elevated in patients with SIRS, sepsis and septic shock [2], and the predictive ability of NGAL in these patients is not so certain [3]. It is unclear, however, whether this predictive relationship is due to the fact that NGAL is produced by neutrophils and is, therefore, a biomarker of inflammation and infection, or whether NGAL in blood and/or urine mostly reflects tubular release. It is also unclear if the type of AKI that develops in SIRS is different from that developing in septic patients.

## Methods

To test these hypotheses, we studied ICU patients with SIRS and oliguria or a 25 μmol/l increase in serum creatinine. We sought to determine whether blood and urine NGAL correlated more closely with CRP or creatinine at the time of enrolment. The strength of the relationship between serum creatinine or CRP and urine and serum NGAL, as well as urinary NGAL corrected for urinary creatinine, was determined using Spearman's rank correlation coefficient.

## Results

We recruited 105 patients between 31 August 2010 and 17 November 2010; 22 of these had an APACHE III diagnosis of sepsis. In nonseptic patients NGAL in blood or urine correlated only weakly with CRP, but a stronger and statistically significant relationship was observed between serum and/or urine NGAL and serum creatinine. A similar strength of relationship was observed between creatinine and NGAL and CRP and NGAL in septic patients, although it failed to reach significance. See Table 1.

**Table 1:** Relationships between NGAL, creatinine and CRP in patients with and without sepsis

		Correlation		
	
	Sepsis (*n *= 22)		No sepsis (*n *= 83)	
	
Variable	Serum Cr	CRP	Serum Cr	CRP
Urinary NGAL	0.345	0.302	0.391	0.057
	(*P *= 0.116)	(*P *= 0.173)	(*P *< 0.001)	(*P *= 0.61)
Urinary NGAL corrected for urinary creatinine	0.311	0.235	0.514	0.070
	(*P *= 0.171)	(*P *= 0.305)	(*P *< 0.001)	(*P *= 0.532)
Serum NGAL	0.244	0.411	0.661	-0.034
	(*P *= 0.274)	(*P *= 0.057)	(*P *< 0.001)	(*P *= 0.763)

## Conclusion

In patients without a diagnosis of sepsis, NGAL is only weakly correlated with CRP and a stronger relationship is observed between NGAL and serum creatinine. This suggests that NGAL is more likely a biomarker of tubular injury or stress than systemic inflammation in these patients. Similar relationships of moderate strength are observed between NGAL in blood/urine and both serum creatinine and CRP in patients with a diagnosis of sepsis. This suggests that different pathophysiological processes may exist in the genesis of septic AKI when compared with inflammatory AKI. Further investigation regarding the natural history of AKI and the clinical and biochemical association of renal biomarkers is warranted.
